# Construction of a human hTERT RPE-1 cell line with inducible Cre for editing of endogenous genes

**DOI:** 10.1242/bio.059056

**Published:** 2022-02-16

**Authors:** Naushin L. Hindul, Amarjot Jhita, Daiana G. Oprea, Tasnim Alamgir Hussain, Oksana Gonchar, Miguel Angel Muro Campillo, Laura O'Regan, Masato T. Kanemaki, Andrew M. Fry, Kouji Hirota, Kayoko Tanaka

**Affiliations:** 1 Department of Molecular and Cell Biology, Henry Wellcome Building, University of Leicester, University Road, Leicester LE1 7RH, UK; 2 Department of Chemistry, Graduate School of Science, Tokyo Metropolitan University, Minamiosawa 1-1, Hachioji-shi, Tokyo, 192-0397, Japan; 3 Department of Chromosome Science, National Institute of Genetics, Research Organization of Information and Systems (ROIS), Yata 1111, Mishima, Shizuoka 411-8540, Japan; 4 Department of Genetics, SOKENDAI, Mishima, Shizuoka 411-8540, Japan

**Keywords:** Endogenous gene editing, hTERT RPE-1, Human culture cell, Inducible Cre recombinase

## Abstract

The human retinal pigment epithelial RPE-1 cell line immortalized with hTERT retains a stable karyotype with a modal chromosome number of 46 and has been widely used to study physiological events in human cell culture systems. To facilitate inducible knock-out or knock-in experiments in this cell line, we have modified the *AAVS1* locus to harbour a DNA fragment encoding ER^T2^-Cre-ER^T2^ fusion protein under regulation of a Tet-On expression system. In the generated cell line, active Cre recombinase was induced by simple addition of doxycycline and tamoxifen to the culture medium. As proof of concept, we successfully introduced an oncogenic point mutation to the endogenous *KRAS* gene locus of this cell line. The cell line will serve as a powerful tool to conduct functional analyses of human genes.

## Abstract (precis)

**Summary:** A near wild-type human hTERT RPE-1 cell line with inducible Cre recombinase integrated at the *AAVS1* was generated for inducible genetic knock-in and knock-out. It facilitates human gene functional studies.

## INTRODUCTION

Cell culture has been an informative experimental technique to conduct functional analyses of genes without interrogating whole organisms. To study the physiological consequences of an individual mutation, the chromosome locus ideally needs to be edited in normal cells that do not carry other genetic abnormalities. Furthermore, to study possible deleterious or transient consequences, the mutation needs to occur conditionally. However, because of the relatively lengthy technical procedures involved, gene function is often probed by overexpressing a recombinant version of the gene of interest in an established cell line. Moreover, in human cell culture systems, many of these cell lines are derived from tumours.

The Cre-LoxP system is a well-established approach to conduct inducible knock-out (KO) or knock-in (KI) experiments (Van Duyne, 2001). Cre is a bacteriophage P1 protein, a site-specific DNA recombinase that recognises a 34 bp DNA sequence called *loxP* (Hoess et al., 1982; Sternberg and Hamilton, 1981). When a DNA fragment containing a segment flanked by repeated *loxP* sites is provided as a substrate, Cre mediates recombination between the two *loxP* sites, excising the intervening DNA segment *in vitro* as well as in cultured mammalian cells and in mice (Abremski and Hoess, 1984; Lakso et al., 1992; Orban et al., 1992; Sauer and Henderson, 1988). To regulate its recombination activity, Cre can be fused with the ligand-binding domain (LBD) of the estrogen receptor (ER) so that it only becomes active when estrogen is provided (Metzger et al., 1995). Tight experimental control of the ER-LBD was enhanced by using mutagenesis to reduce its affinity towards the natural ligand, estrogen, which may be available in the cell, while increasing its affinity towards a synthetic ligand such as 4-hydroxy tamoxifen (4-OHT). The most commonly used variant for inducible gene editing is ER^T2^, which contains a G400*V*/M543A/L544A triple mutation of the human ER-LBD and exhibits a highly selective affinity towards 4-OHT (Feil et al., 1997).

In mouse models, a wide range of Cre lines are available for conditional KO and KI in a tissue or developmental-stage specific manner (Wang, 2009). However, in human cell culture systems, Cre is typically provided in the form of a plasmid or a viral construct (Sengupta et al., 2017). Plasmid transfection or viral transduction, though, adds an extra step to the experimental procedure and can introduce additional experimental variations to the outcome.

To circumvent this issue, we decided to generate an hTERT RPE-1 stable cell line where ER^T2^-Cre-ER^T2^ is integrated at the *AAVS1* locus under the regulation of a Tet-On system. The hTERT RPE-1 cell line was derived from a normal human retinal pigment epithelial (RPE) cell line, RPE-340, which was immortalised by the human telomerase reverse transcriptase subunit (hTERT) (Bodnar et al., 1998; Jiang et al., 1999). As hTERT RPE-1 features a near-diploid karyotype with a modal chromosome number of 46 and lacks transformed phenotypes, the cell line has been frequently used to study the normal physiological function of human genes. Importantly, its stable and normal karyotype allows targeted genome editing. We chose the *AAVS1* locus for ER^T2^-Cre-ER^T2^ integration as it was shown to be a ‘safe harbour’ to insert and express transgenes (Smith et al., 2008), and effective *AAVS1* CRISPR/Cas9 targeting constructs are available (Cong et al., 2013; Mali et al., 2013; Natsume et al., 2016). As proof-of-concept, the generated cell line successfully excised a LoxP cassette integrated at the *KRAS* gene locus after 48 h of doxycycline and tamoxifen treatment. Hence, the inducible hTERT RPE1 ER^T2^-Cre-ER^T2^ cell line will serve as a powerful tool for the scientific community to conduct functional analyses of human genes.

## RESULTS

### Design of ER^T2^-Cre-ER^T2^ *AAVS1* integration plasmid

**Fig. 1.**
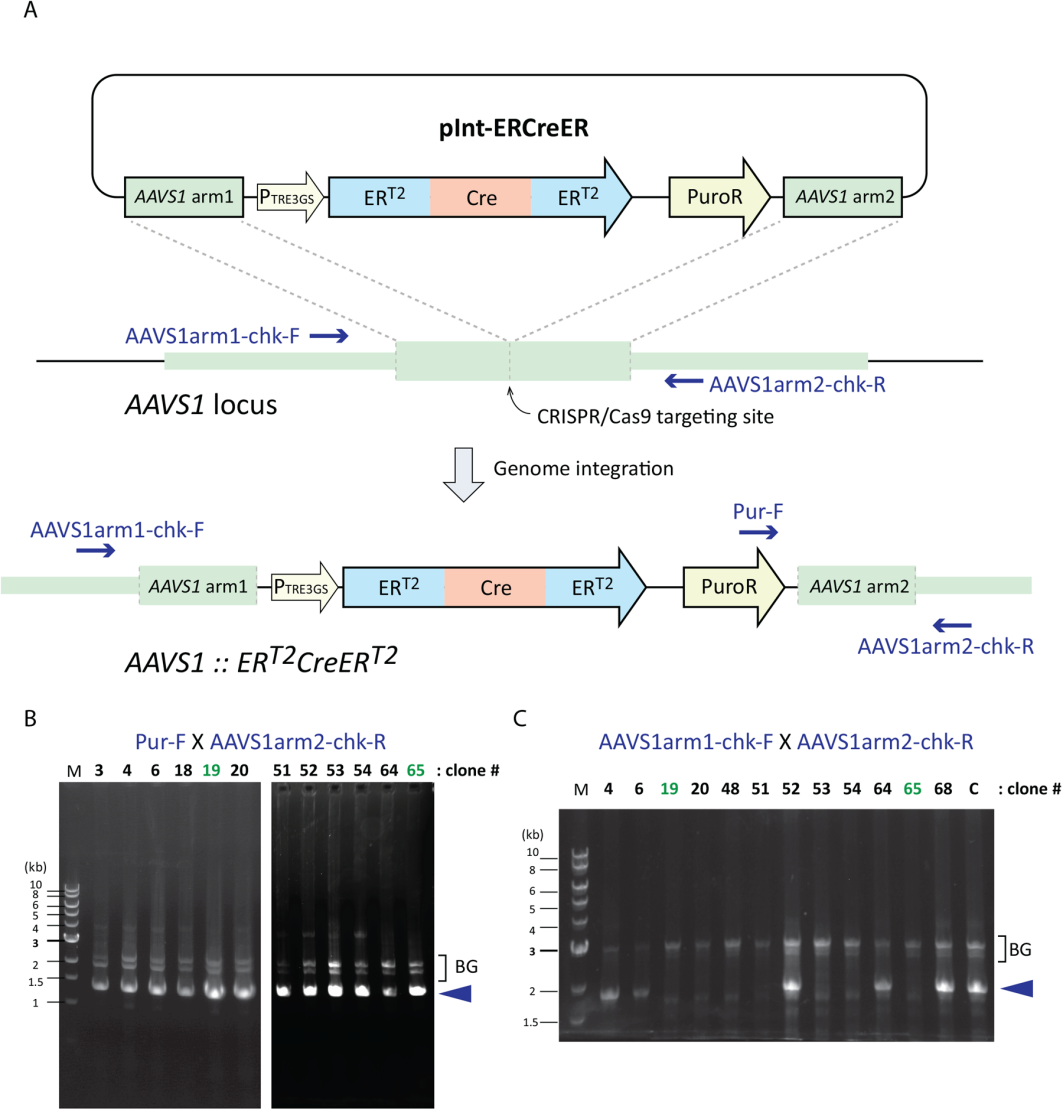
**Construction of hTERT RPE-1 ER^T2^-Cre-ER^T2^ cell line.** (A) A schematic diagram of the integration donor plasmid pInt-ERCreER and the targeted *AAVS1* locus. Locations of the primers used to genotype the integration event are indicated in blue. (B) Representative genome PCR results using primers Pur-F and AAVS1arm2-chk-R. Successful integration of the ER^T2^-Cre-ER^T2^ cassette into *AAVS1* locus of at least one of the chromosomes yields a 1330 bp PCR product (indicated by a blue arrowhead). Numbers indicated for each lane represent clones isolated through the puromycin resistance screening. Clones 19 and 65 (shown in green) were further analysed in this study. BG indicates background bands. (C) Representative genome PCR results using primers AAVS1arm1-chk-F and AAVS1arm2-chk-R. This pair of primers amplifies an 1836 bp fragment (indicated by a blue arrowhead) if the wild-type unedited *AAVS1* locus is present. The result indicates that clones 19, 20, 48, 51, 53, 54 and 65 do not retain the wild-type *AAVS1* locus. Together with the result from panel B, we concluded that these clones carry the ER^T2^-Cre-ER^T2^ cassette as a homozygous insertion at the *AAVS1* locus. Clones 19 and 65 were further analysed for Cre expression and activity in Figs 2 and 3. BG indicates background bands.

A possible drawback to generate a cell line where a gene encoding the Cre recombinase is stably integrated into the genome is that a basal level of Cre expression may cause DNA strand breaks that are cytotoxic or evoke the DNA damage checkpoint (Schmidt et al., 2000; Silver and Livingston, 2001). To minimise this effect, we implemented two approaches. First, we fused ER^T2^ at both the N- and C-terminus of Cre as this double fusion of Cre was shown to have the least basal activity in the absence of 4-OHT (Casanova et al., 2002; Zhang et al., 1996). Second, to repress gene expression of ER^T2^-Cre-ER^T2^ in the uninduced state, we used a conditional Tet-On system that has minimal transcriptional activity in the absence of doxycycline (Das et al., 2016; Loew et al., 2010; Zhou et al., 2006). The DNA fragment, encoding ER^T2^-Cre-ER^T2^ fusion protein, under regulation of the Tet-On system, together with a puromycin resistant gene (encoding puromycin *N*-acetyltransferase, PAC) (Vara et al., 1986), was flanked by *AAVS1* homologous arms to generate the integration plasmid pInt-ERCreER (Fig. 1A).

**Fig. 2.**
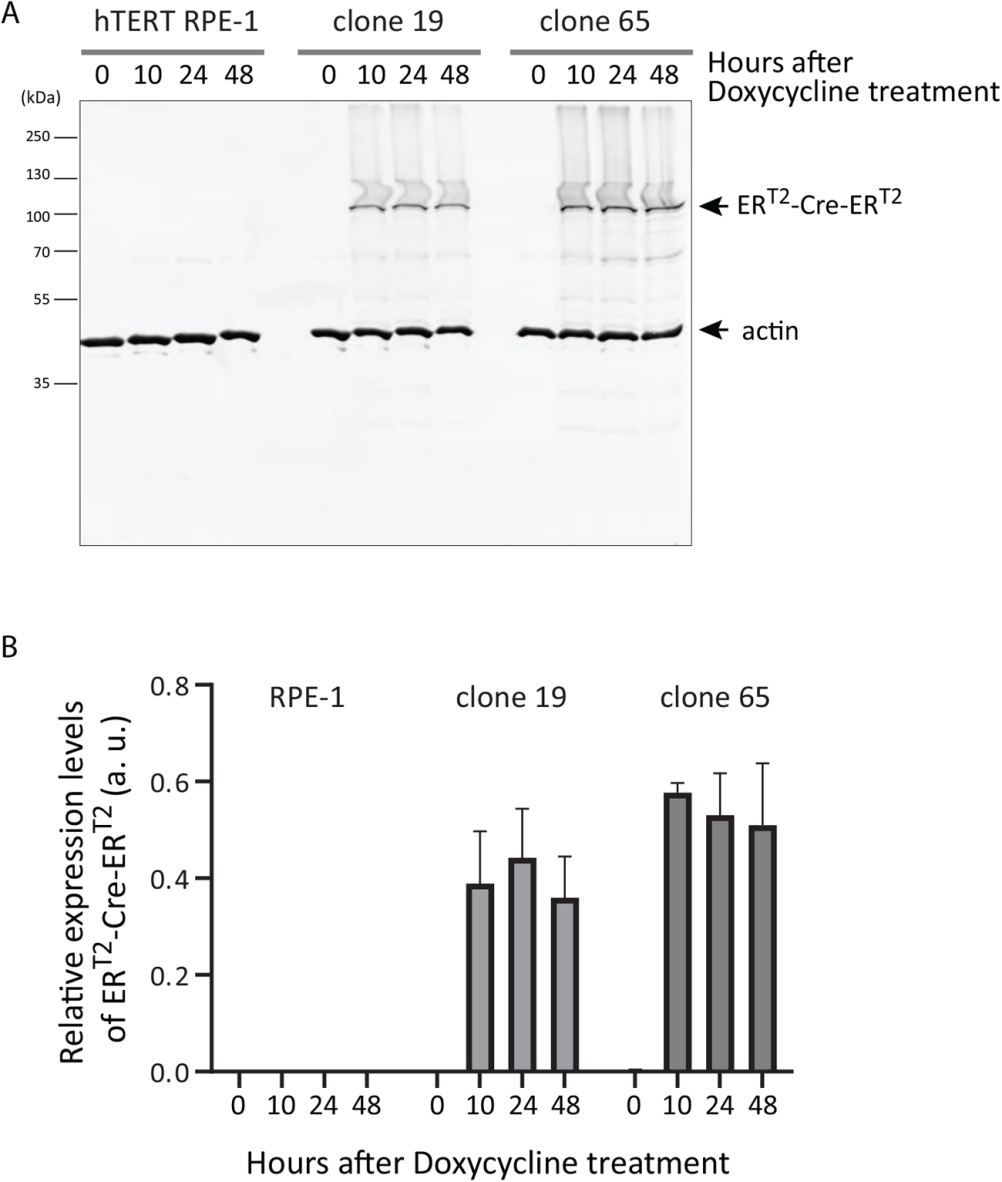
**Expression of ER^T2^-Cre-ER^T2^ is induced within 10 h after doxycycline treatment.** hTERT RPE-1 ER^T2^-Cre-ER^T2^ clones 19 and 65 were treated with doxycycline (1 µg/ml) and the expression levels of ER^T2^-Cre-ER^T2^ at time 0, 10, 24 and 48 h after the treatment were analysed by western blotting. (A) A representative result of the parental hTERT RPE-1, clone 19 and clone 65. (B) Three biological replicates of the western blotting were quantitated using actin as an internal control. Mean and SD values are presented.

**Fig. 3.**
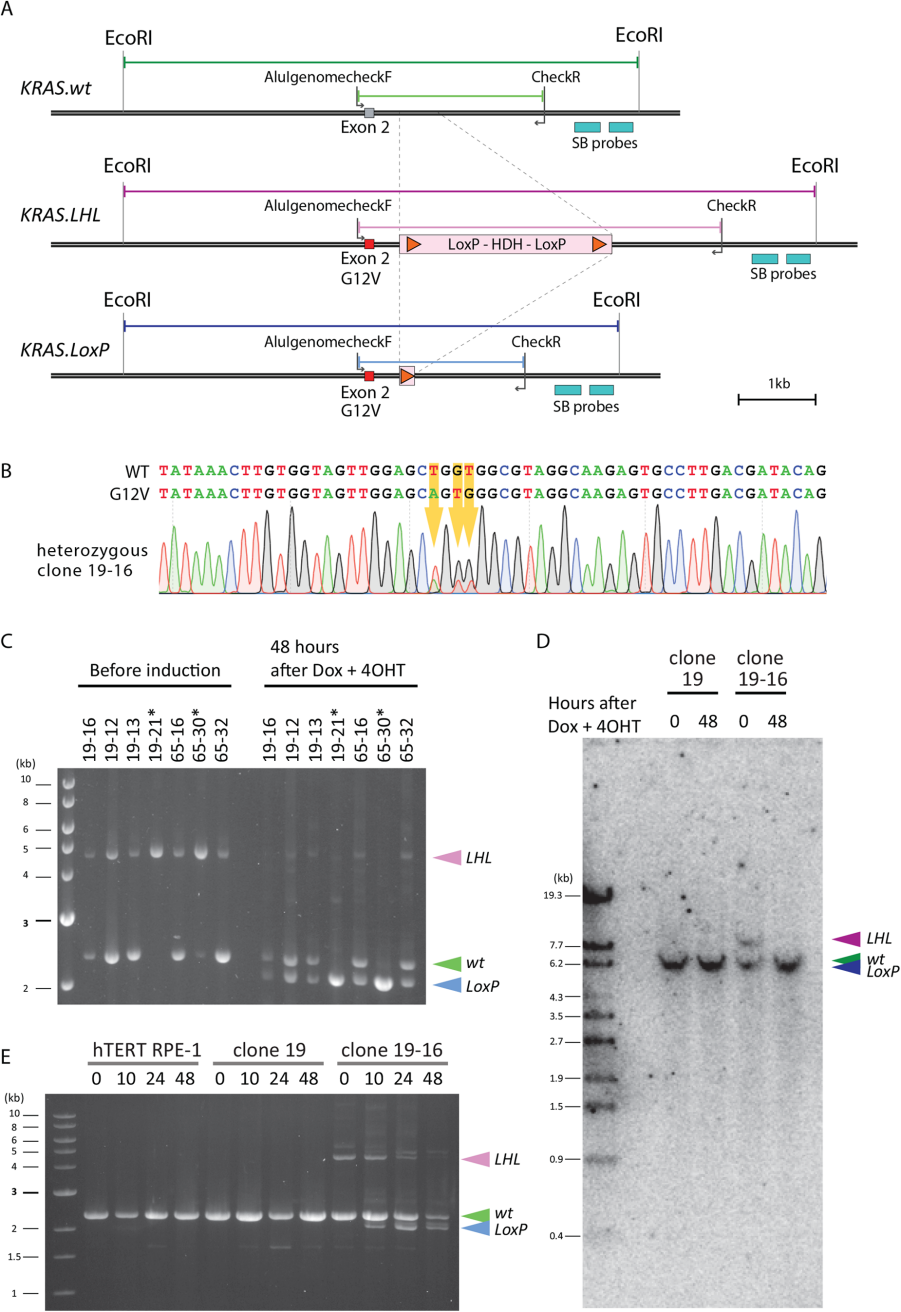
**Cre-LoxP excision of the targeted chromosome in 48 h after doxycycline and 4-OHT treatment.** (A) A schematic diagram of a proof-of-principle test case, chromosome editing of the *KRAS* gene locus. A DNA fragment encoding histidinol dehydrogenase (HDH), flanked by LoxP sites (LoxP-HDH-LoxP, LHL) was inserted into intron 2 of *KRAS* gene to create *KRAS.LHL* locus. Active Cre is expected to excise the LHL cassette, leaving one copy of LoxP site, to generate *KRAS.LoxP* allele. A pair of primers, AlugenomecheckF and CheckR, is indicated in the diagram. Two probes used for the Southern blotting are indicated as ‘SB probes’ and *EcoRI* sites used for Southern blotting are indicated in the diagram. (B) Some of the clones with the LoxP cassette insertion also carried the *KRAS.G12V* mutation. The genome isolated from clone 19-16, which has a heterozygous integration of the ER^T2^-Cre-ER^T2^ cassette, was sequenced using the primer AlugenomecheckF. Three substitutions of the nucleic acids resulting in G12V mutation are indicated with orange arrows together with the sequencing chromatogram. (C) A typical example of successful excision of the LHL cassette 48 h after doxycycline (1 µg/ml) and 4-OHT (0.5 µM) exposure in seven independent clones. Clones 19-16, 19-12, 19-13 and 19-21 were generated by integrating the LHL cassette into the *KRAS* locus of clone 19 and clones 65-16, 65-30 and 65-32 were generated in the same way but using clone 65. Among these clones, clones 19-21 and 65-30 have *KRAS.LHL* as a homozygous insertion. PCR genotyping was conducted using primers AlugenomecheckF and CheckR. The PCR reaction produced the expected products of 4621 bp for *KRAS.LHL*, 2370 bp for *KRAS.wt* and 2115 bp for *KRAS.LoxP*. (D) Southern blotting to confirm the successful LHL excision 48 h after doxycycline and 4-OHT treatment. Genomes from clone 19 and clone 19-16 were digested by *EcoRI* and analysed by Southern blotting using a cocktail of two probes indicated in the diagram A (SB probes). Signals were detected at the expected size of 8797 bp for *KRAS.LHL*. For *KRAS.wt* and *KRAS.LoxP*, the expected sizes were 6546 bp and 6291 bp, respectively, and these two bands were not resolved in the agarose gel. Nonetheless, the *KRAS.LHL* signal became undetectable in clone 19-16 (time 48) sample, and therefore we concluded that LHL excision was successful 48 h after exposure to doxycycline and 4-OHT. (E) Time-course profile of the LHL excision. Parental hTERT RPE-1, clone 19 and clone 19-16 were exposed to doxycycline (1 µg/ml) and 4-OHT (0.5 µM), and samples collected at 0, 10, 24 and 48 h after treatment. Genome PCR was conducted using primers AlugenomecheckF and CheckR. A PCR product of *KRAS.LoxP* was detectable 10 h after the start of the treatment. By 48 h, most of the *KRAS.LHL* signal disappeared and the signal intensity of *KRAS.wt* and *KRAS.LoxP* became comparable.

### Integration of ER^T2^-Cre-ER^T2^ at the *AAVS1* locus in hTERT RPE1 cells

hTERT RPE1 cells were transfected with pInt-ERCreER together with an *AAVS1* targeting CRISPR/Cas9 construct (Addgene, plasmid #72833) (Natsume et al., 2016). To isolate integrated clones, first puromycin screening was carried out. hTERT RPE1 cells are resistant to puromycin at a concentration within the range of 0.2–5 µg/ml because hTERT was originally introduced to the ancestral RPE cells with a plasmid pGRN145 (Weinrich et al., 1997) (ATCC, MBA-141) carrying both the Hygromycin B phosphotransferase and PAC genes (Bodnar et al., 1998). However, after conducting preliminary kill-curve experiments, we found that hTERT RPE1 cells can be screened using 6–8 µg/ml of puromycin.

69 clones resistant to puromycin (6–8 µg/ml) were selected and their genomes were isolated. Successful integration into at least one of the *AAVS1* loci was confirmed for 30 out of the 69 clones by PCR genotyping using a pair of primers spanning one of the integration sites (Fig. 1B). Out of these, 12 clones were further examined for retention of the wild-type unedited *AAVS1* locus, and five clones were found to be positive (Fig. 1C). We considered them to have a heterozygous integration of the ER^T2^-Cre-ER^T2^ cassette, while the remaining seven clones (clones 19, 20, 48, 51, 53, 54 and 65 in Fig. 1C) were homozygous for ER^T2^-Ce-ER^T2^ at *AAVS1*. Of the seven homozygous clones, the *AAVS1* loci of clones 19 and 65 were sequenced, and ER^T2^-Cre-ER^T2^ confirmed to be free of mutations in both clones (Fig. S1).

### Doxycycline dependent expression of ER^T2^-Cre-ER^T2^ in hTERT RPE1 cells

hTERT RPE1 clones 19 and 65 were further examined for the expression profile of ER^T2^-Cre-ER^T2^ upon doxycycline treatment. Cell extracts were prepared from these two clones as well as the parental hTERT RPE-1 cells at 0, 10, 24 and 48 h after doxycycline addition (1 µg/ml) to the media. Expression of ER^T2^-Cre-ER^T2^ was detected by western blotting using an anti-ERα antibody (Fig. 2A). At 0 h a band was not detected in any of the three samples, whereas after 10 h a band appeared at the expected size of ∼100 kDa in samples from clones 19 and 65 but not the parental cells; the intensity of this band stayed unchanged for the following 38 h. Quantification of ER^T2^-Cre-ER^T2^ expression in three biological replicates revealed that doxycycline induced the maximum level of ER^T2^-Cre-ER^T2^ expression in both clones 19 and 65 by 10 h after induction (Fig. 2B). The expression level of ER^T2^-Cre-ER^T2^ was slightly higher in clone 65 than in clone 19, albeit by a relatively small amount.

### Inducible chromosome editing within 48 h after doxycycline and 4-OHT treatment

Having confirmed doxycycline-dependent ER^T2^-Cre-ER^T2^ expression, we introduced a LoxP cassette into the genome of clones 19 and 65 to examine whether the induced Cre fusion protein was able to excise the LoxP cassette upon doxycycline and 4-OHT treatment. For this purpose, a 2.54 kb LoxP cassette was integrated into intron 2 of the *KRAS* gene locus in clones 19 and 65 as described in the Materials and Methods (Fig. 3A). The LoxP cassette integration plasmid (pKH-His-DA-Ap) included the *KRAS* exon 2 as a part of the 5′ homologous arm, and we introduced an oncogenic mutation G12V into exon 2. Therefore, some of the clones that successfully integrated the LoxP cassette also carried the G12V mutation (Fig. 3B). Phenotypic studies of the resultant *KRAS.G12V* cell line will be reported elsewhere.

To examine consistency and robustness of ER^T2^-Cre-ER^T2^ – mediated LoxP cassette excision, four independent clone 19-derivatives (19-16, 19-12, 19-13 and 19-21) and three independent clone 65-derivatives (65-16, 65-30 and 65-32) were analysed for the *KRAS* alleles before and after exposure to doxycycline (1 µg/ml) and 4-OHT (0.5 µM) for 48 h. The genomes were isolated and analysed by PCR using a pair of primers, AlugenomecheckF and CheckR (Fig. 3C). Before the doxycycline and 4-OHT treatment, homozygous clones, 19-21 and 65-30, gave a single PCR product of 4.6 kb, corresponding to the *KRAS.LHL* allele where the LoxP cassette is inserted, whereas heterozygous clones, 19-16, 19-12, 19-13, 65-16 and 65-32, gave an additional product of 2.4 kb, corresponding to the *KRAS.wt* allele (Fig. 3C). 48 h after the treatment, the 4.6 kb PCR product became undetectable or was substantially reduced in all samples. For heterozygous clones, the level of the PCR product from *KRAS.LoxP* allele, which was produced as the result of LoxP cassette excision, became comparable to the level of PCR product from the *KRAS.wt* allele (Fig. 3C,D). For the clone 19-16, we conducted genomic Southern blotting to confirm the excision (Fig. 3E). Time-course analysis of clone 19-16 showed that LoxP cassette excision was detectable at 10 h after doxycycline and 4-OHT treatment and completed by 48 h post-treatment (Fig. 3E). From these results, we conclude that the hTERT RPE-1 ER^T2^-Cre-ER^T2^ cell line allows us to conduct inducible gene editing and that excision of the LoxP cassette can be achieved within 48 h of addition of doxycycline and 4-OHT to the medium.

### Cre expression causes a transient but reversible DNA damage response

**Fig. 4.**
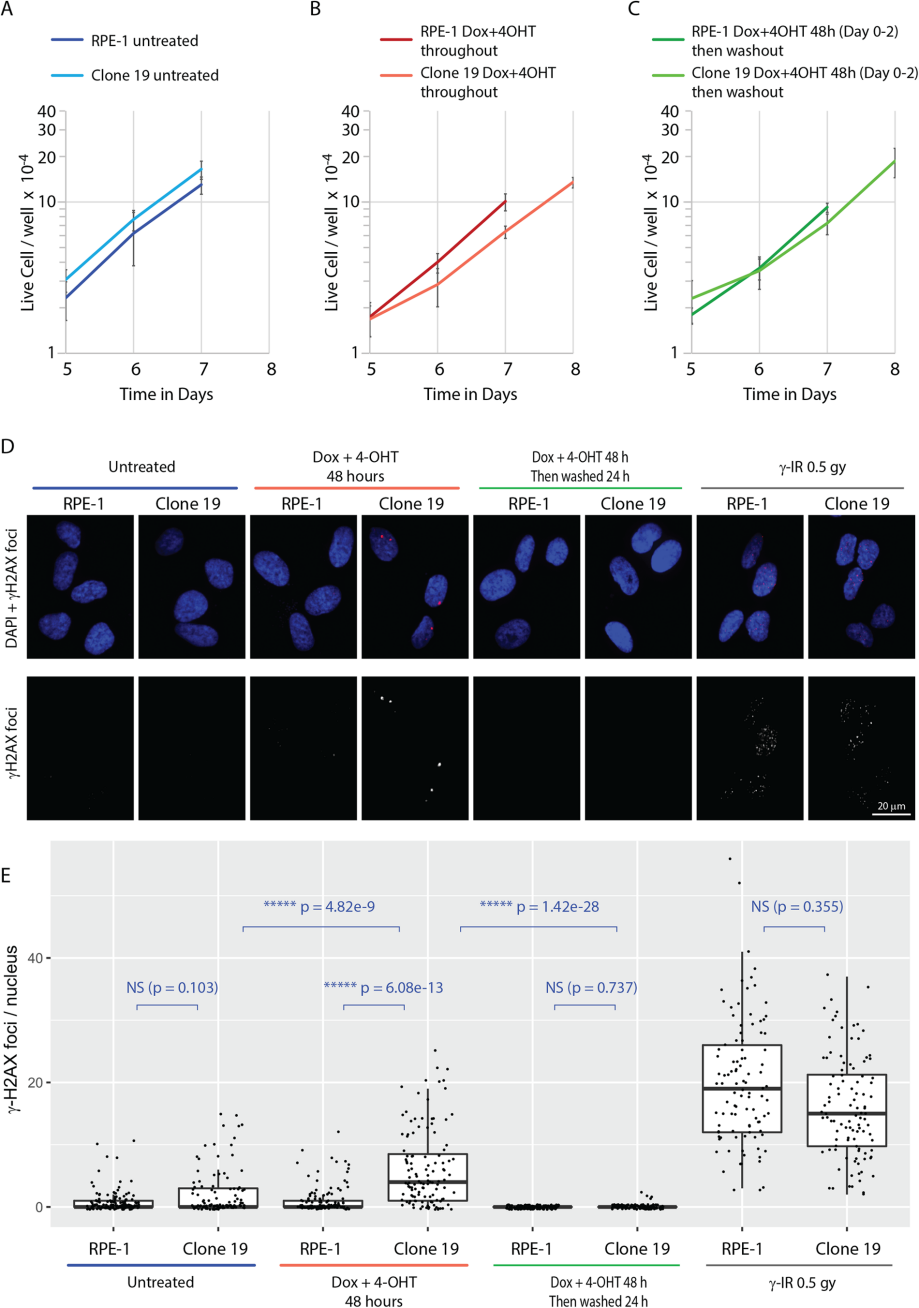
**The physiological impact of expression of ER^T2^-Cre-ER^T2^ in the clone 19.** (A–C) Proliferation rates of RPE1 and clone 19 cells with or without doxycycline and 4-OHT treatment. A relatively small number of cells (2×10^3^ cells) were seeded per well in a six-well plate to allow observation of proliferation for up to 8 days. The panels show the proliferation rates from day 5. The cell count data for the whole course of the experiment is presented in the Fig. S2. All graphs have a logarithmic y-axis. (A) Proliferation rates of RPE1 and clone 19 cells without doxycycline and 4-OHT treatment. (B) Proliferation rates of RPE1 and clone 19 cells cultured in the presence of doxycycline and 4-OHT throughout the experiment. (C) Proliferation rates of RPE1 and clone 19 cells treated for 48 h with doxycycline and 4-OHT before replacement into fresh media. For the clone 19 samples treated with doxycycline and 4-OHT, cell counts were made for an additional day to reveal growth recovery of these samples. All experiments, *n*=3; error bars represent SD. (D) Formation of γ-H2AX foci upon expression of ER^T2^-Cre-ER^T2^. RPE-1 and Clone 19 cells were subject to the following treatments: incubation for 48 h with doxycycline and 4-OHT, incubation for 48 h with doxycycline and 4-OHT, which were then removed for 24 h before fixation, and γ-irradiation (0.5 Gy) 30 min before fixation. Untreated cells are included as a reference. γ-H2AX foci formation was detected by immunofluorescence microscopy as described in the Materials and Methods. Upper panels show merged images of γ-H2AX foci and DNA. Scale bar: 20 µm. (E) Summary of γ-H2AX foci formation results presented in (D). 100–150 nuclei were counted for each sample and the data presented using the boxplot format (RStudio) where median, and first and third quartiles are indicated using a box. Individual data points are also indicated. The data were analysed by Kruskal–Wallis test and pairwise comparisons conducted by Dunn’s test. NS, not significant; **P<0.01, ***P<0.001, ****P<0.0001, *****P<0.00001.

To evaluate a possible adverse effect of Cre recombinase expression in the cell line we have generated, we examined the proliferation rate and the DNA damage response of the parental RPE-1 and clone 19 cells under the same conditions used for genome editing. Cells were treated for 48 h with doxycycline and 4-OHT, which were then washed out, and cell proliferation rate and γ-H2AX foci formation, a marker of DNA damage, were monitored (Fig. 4; Fig. S2).

We first seeded a relatively small number of cells in a culture well so that we could observe proliferation for 7–8 days without cell passaging. The relatively low density of the cells of this condition resulted in a slower proliferation rate during the first 5 days after seeding before a more exponential rate of growth (Fig. S2A,B). Under this condition, both untreated RPE-1 and clone 19 cells showed a comparable proliferation profile (Fig. 4A). For clone 19, doxycycline and 4-OHT treatment, either for 48 h or continuously, resulted in a reduction in cell number compared to the untreated clone 19 cells on Day 6 (Fig. S2B). The proliferation rates between day 5 and 6 of these samples are reduced compared to the corresponding RPE-1 samples, indicating a potential DNA damage response (Fig. 4B,C). However, these cells showed a recovery of proliferation after day 6. Recovery of proliferation of the continuously treated cells indicates that the degree of DNA damage caused by Cre induction is relatively mild. The washout sample showed no detectable impairment in cell proliferation after further passaging (data not shown).

We next monitored the occurrence of DNA double-strand breaks (DSBs) by staining for γ-H2AX foci (Rogakou et al., 1998). Both untreated RPE-1 and clone 19 cells showed a low and comparable number of γ-H2AX foci, whereas upon 48 h treatment with doxycycline and 4-OHT clone 19 showed an increased number of γ-H2AX foci compared to RPE-1 cells (Fig. 4D,E). Not unexpectedly, this indicates that the expressed Cre recombinase generates a number of DSBs in clone 19 cells. However, these foci disappeared within 24 h after removal of doxycycline and 4-OHT, indicating that the DSB formation in clone 19 under the conditions used for gene-editing is transient.

Taken together, we conclude that the amount of Cre induced for gene-editing causes only a transient increase in DNA damage without substantially affecting cell proliferation, and that clone 19 is therefore a useful tool for functional gene analyses in human cells.

## DISCUSSION

It has been well recognised that many genes are functionally conserved between humans and mice, and mouse models have made valuable contributions to understand mechanisms of human diseases. However, important differences between these two organisms also have become evident in recent years (Uhl and Warner, 2015). Therefore, it is vital to keep developing experimental tools for human cell culture systems that allow human gene function studies. Unlike mouse models where mouse embryonic fibroblasts can be produced from mice of a defined and purposely engineered genetic background, immortalised human cell lines are mostly derived from tumour tissues of varying genetic backgrounds with unstable karyotypes. Hence, establishment of the hTERT-mediated immortalisation protocol and subsequent generation of the hTERT RPE-1 cell line have made an immense impact on the field, as these developments have allowed researchers to study normal, or ‘near wild type’, human cells that retain a stable karyotype, cell cycle checkpoints and contact inhibition (Bodnar et al., 1998; Jiang et al., 1999).

In this study, we generated the pInt-ERCreER plasmid, which, together with an *AAVS1*-targeting CRISPR/Cas9 construct, allows integration of an ER^T2^-Cre-ER^T2^ cassette at the *AAVS1* locus of human cell lines. We observed that the integration efficiency in hTERT RPE-1 cells was reasonably high (30/69 clones), and multiple clones were found to carry the ER^T2^-Cre-ER^T2^ cassette as a homozygous insertion. This was surprising as hTERT RPE-1 cells are considered to retain a relatively low homologous recombination activity (Katoh et al., 2017; Miyamoto et al., 2015). We reasoned that the unexpected high integration rate may be due to insertion at the *AAVS1* locus, which is a hotspot for adeno-associated virus (AAV) integration (Kotin et al., 1992) and retains a DNA segment that prevents the spread of heterochromatin (Ogata et al., 2003). Although AAV integration occurs through a non-homologous recombination pathway, the somewhat ‘open’ nature of the chromosome structure in this region may also be more susceptible for homologous recombination. As the pInt-ERCreER plasmid performed successfully in the ‘difficult’ hTERT RPE-1 cells, we expect it to be even more effective in other human cell lines.

During the process of generating hTERT RPE-1 ER^T2^-Cre-ER^T2^ cells, we managed to utilise puromycin resistance to integrate the ER^T2^-Cre-ER^T2^ cassette. Therefore, other useful selection reagents such as neomycin, blasticidin and histidinol are still available for gene editing in this cell line. As proof-of-principle for use of this cell line for gene editing, we used histidinol dehydrogenase gene as a selection marker to insert a LoxP cassette in intron 2 of the *KRAS* gene locus. The LoxP cassette was then successfully excised within 48 h after Cre induction, confirming that using the generated hTERT RPE-1 ER^T2^-Cre-ER^T2^ cell line, we can conduct inducible gene editing by simply adding doxycycline and 4-OHT to the culture medium.

The physiological impact of the Cre expression in this cell line was evaluated by analysing the cell proliferation rate and DNA damage response. The results showed that the overall fitness of the hTERT RPE-1 ER^T2^-Cre-ER^T2^ cell line is comparable to the parental cell line, and the condition used to induce the Cre-mediated genome editing causes a minor reversible DNA damage response. Together, the hTERT RPE-1 ER^T2^-Cre-ER^T2^ cell line is expected to exhibit minimum artefact when utilised for human genome editing experiments.

Our preliminary result showed that, in the *KRAS.G12V* heterozygous cell line, the inserted LoxP cassette did not block the transcription or splicing of the *KRAS.G12V*, and therefore, Kras.GV protein was expressed regardless of the Cre-induced LoxP cassette excision. Hence, the case demonstrates that the inducible genome alteration does not always result in inducible protein expression. However, if the LoxP cassette insertion is to disrupt gene transcription or splicing, we would be able to express the encoded protein in an inducible manner using this cell line.

This hTERT RPE-1 ER^T2^-Cre-ER^T2^ cell line is versatile in gene editing. In addition to conditional gene deletion, we can effectively introduce a point mutation to a gene using a selection marker, which is subsequently removed to leave the gene allele with minimum alteration. Furthermore, inducible protein expression is possible by placing a LoxP cassette appropriately. The hTERT RPE-1 ER^T2^-Cre-ER^T2^ cell line will therefore facilitate future functional studies of human genes and genome.

## MATERIALS AND METHODS

### Plasmid cloning

pInt-ERCreER was generated by modifying pMK240 (Natsume et al., 2016). A DNA fragment encoding a fusion protein of the ligand-binding domain of human estrogen receptor with T2 mutation (ER^T2^), Cre recombinase and ER^T2^ was synthesised (Integrated DNA Technologies, Inc.) and inserted at f Mlu I –Bgl II sites at the multi-cloning site of the plasmid using In-Fusion HD EcoDry Cloning Plus (Takara Bio). Insertion of a LoxP cassette at *KRAS* locus of the chromosome was mediated by two plasmids, pX330-kras_CRISPR and pKH-His-DA-Ap_kras_integration. pX330-kras_CRISPR is a derivative of pX330 (Addgene, #42230) (Cong et al., 2013), where the guide RNA sequence for the targeted *KRAS* gene locus, 5′ GTATTTCAGAGTTTCGTGAG 3′, is inserted. pKH-His-DA-Ap_*KRAS*_integration plasmid contains a DNA fragment encoding histidinol dehydrogenase under PGK promoter, which is flanked by a pair of LoxP sites. This LoxP cassette is sandwiched by *KRAS* gene homologous arms. The left (5′) arm spans position 9447 to 10951 of chromosome 12, carrying an oncogenic G12*V* mutation in exon2, and the right (3′) arm spans position 11399 to 12753 of chromosome 12.

### Cell culture and transfection

To culture hTERT RPE-1 cells (ATCC, CRL-4000), DMEM/F-12 (Gibco, #31331-028) containing 0.5% sodium bicarbonate, supplemented with 10% foetal bovine serum (Gibco, #10500-064), 1% (v/v) Penicillin-Streptomycin (Gibco, #15140-122) and 10 µg/ml Hygromycin B Gold (InvivoGen, #ant-hg-1) was routinely used. Cells were cultured at 37°C, 5% CO_2_. Transfection was conducted using Lipofectamine 3000 Reagent (Invitrogen, #L3000001) following the manufacturer's instruction. To select clones with ER^T2^-Cre-ER^T2^ integration at *AAVS1* locus, puromycin (InvivoGen, #ant-pr-1) was added to the media at a final concentration of 6–8 µg/ml. To select clones with a LoxP cassette integration at *KRAS* gene locus, L-histidinol dihydrochloride (Sigma-Aldrich, #H6647) was added to the media at a final concentration of 1.35–1.5 mg/ml. For doxycycline treatment, doxycycline (Alfa Aesar, #J60422) was dissolved in H_2_O at 1 mg/ml and used at a final concentration of 1 μg/ml in the medium. For 4-OHT treatment, 4-OHT (Merck, #H6278) was dissolved in ethanol at 1 mM and used at a final concentration of 0.5 μM in the culturing medium.

### PCR based genotyping

GeneJET Genomic DNA Purification Kit (Thermo Fisher Scientific, #K0721) was used to isolate genome DNAs from isolated clones. To confirm integration of ER^T2^-Cre-ER^T2^ at *AAVS1* locus, primers Pur-F (5′ TTCTACGAGCGGCTCGGCTTCACCGTCA 3′) and AAVS1arm2-chk-R (5′ GTTGGAGGAGAATCCACCCAAAAGGCAGCC 3′) were used. To examine the presence of *AAVS1* wild-type (unedited) locus, primers AAVS1arm1-chk-F (5′ CGCCTCTGGCCCACTGTTTCCCCTTCCC 3′) and AAVS1arm2-chk-R (5′ GTTGGAGGAGAATCCACCCAAAAGGCAGCC 3′) were used. To examine the structure of *KRAS* locus, a pair of primers AlugenomecheckF (5′ TCATTACGATACACGTCTGCAGTCAACTGG 3′) and CheckR (5′ GTTCTCCTGCCTTTCTTACAGTTTAACTACA 3′) were used. A typical PCR reaction mix was prepared in 50 µl reaction scale by assembling the following components: 3 µl of extracted genome DNA (1–15 ng/µl), 1.5 µl of each primer (10 µM), 1.5 µl of dNTPs (10 mM each), 1 µl of KAPA HiFi DNA polymerase (Roche, #KK2101), 10 µl of 5 x KAPA HiFi buffer (Roche, #KK2101) and 31.5 µl H_2_O. The PCR condition was set as follows; 30 s denaturation at 98°C, 35 cycles of 10 s 98°C, 10 s 68°C, 150 s 72°C and 5 min 72°C.

### Western blotting

For immunoblotting of ER^T2^-Cre-ER^T2^ expression, cells were lysed in 3x Laemmli buffer (240 mM Tris pH 6.8, 6% SDS, 30% glycerol, 2.24 M β-mercaptoethanol, 0.06% Bromophenol Blue) (Laemmli, 1970), and samples were heat-denatured at 95°C for 5 min. Proteins were resolved by SDS-PAGE and transferred to 0.2 μm nitrocellulose membranes (Bio-Rad, #1620112), and the membranes were blocked with a blocking buffer (Li-Cor Intercept Blocking Buffer TBS, #927-60001). The ER^T2^-Cre-ER^T2^ was detected with an anti-ERα antibody (Santa Cruz Biotechnology, #sc-8002, 1/500 dilution), and an anti-actin antibody (Life Technologies, #MA1-744, 1/2000 dilution) was used to detect actin as a loading control. Goat anti-mouse antibody (Li-Cor, #926-68020) was used as a secondary antibody, and membrane images were acquired by Odyssey Infrared Imaging System (Li-Cor Biosciences). The protein levels were quantified using Image Studio software (Li-Cor Biosciences). Fig. 2B presents the results deduced from three biological replicates, analysed by Prism software (GraphPad).

### Southern blotting

Southern blotting was conducted essentially as described previously (Senmatsu et al., 2021) to check the chromosome structure of *KRAS* gene locus. Genomes were isolated by DNAzol reagent (Thermo Fisher Scientific, #10503027) following the manufacturer's instruction. 10 µg of sample genome DNA was digested by *Eco*RI, run on an agarose gel (0.8%) and transferred to a nitrocellulose membrane (Biodyne B, PALL, NY, USA). Two radioactive probes for two regions of *KRAS* gene were prepared as follows. First, two *KRAS* genome regions were amplified by PCR. One was amplified with a pair of PCR primers 5′ GGTAAAATTTGGTGGAAGAGGAAAAGTCTC 3′ and 5′ CAGAACACTAAAGATGAAACAAACCAATCC 3′ and the other using PCR primers 5′ CTCTGAGACCAAGTTAAGTAGAATTTGCAC 3′ and 5′ TGCCCTAATAACGAGGTATTTCATTATCTC 3′. The generated *KRAS* genome fragments were cloned separately in a pBluescript plasmid, from which two PCR fragments were amplified and used as templates for random-primer labelling (GE Healthcare) with [α-^32^P]-dCTP (PerkinElmer, MA, USA). Hybridization was performed in a buffer [1% BSA, 7% SDS, 0.5M Na_2_HPO_4_ (pH 7.4), 1mM EDTA] at 62°C for 12h, and extensively washed with wash buffer [1% SDS, 1mM EDTA, 0.04M Na_2_HPO_4_ (pH 7.4)]. Signal was detected by a phosphor imager (FLA7000, Fuji film, Tokyo).

### Cell proliferation assays

Cell proliferation rates were examined by growing cells in a well of a six-well plate (TPP, #92006). On Day 0, 2×10^3^ cells were seeded in each well, and 2 ml media was provided. Presumably, because the starting cell density was low, cell growth became clearly detectable only on day 5–6. This condition allowed us to confirm the growth recovery of clone 19 on day 7–9 after cells were treated with doxycycline (1 μg/ml) and 4-OHT (0.5 μM). On the day of the measurement, the media in the well was removed, and the cells were rinsed by modified Hanks' balanced salt solution (Sigma-Aldrich, #H6648). Cells were trypsinised by 500 µl of 0.25% Trypsin-EDTA solution (Merck, #T4049) and then mixed with 500 µl of the media. 40 µl of the cell suspension was mixed with 0.4% trypan blue solution (Sigma-Aldrich T8154). The stained cells were counted using CellDrop (DeNovix).

### Quantification of γ-H2AX foci

Cells were seeded in a well of a six-well plate (TPP, #92006), each well containing an acid-treated glass coverslip and 3 ml media. Cells were either untreated or treated with doxycycline (1 μg/ml) and 4-OHT (0.5 μM). As a control sample, cells were γ-irradiated at a dose of 0.5 Gy using Xstrahl RS320. Cells were fixed with 3.7% paraformaldehyde (PFA) in phosphate-buffered saline (PBS) for 10 min at room temperature (RT). PFA was removed by washing the cells with PBS three times, and cells were permeabilised in 0.5% (v/v) Triton X-100 and 0.5% (w/v) saponin in PBS for 10 min at RT. After washing the cells three times in PBS, the remaining PFA was quenched in 1 mg/ml of NaBH_4_ solution for 4 min. The sample was rinsed once with PBS and then treated with 0.1 M glycine in PBS for 1 h at RT. Cells were then rinsed in PBS and blocked in 1% (w/v) BSA in PBS for 1 h at RT. Anti-phospho-histone H2A.X antibody (clone JBW301, Millipore #5636) was diluted to 2 µg/ml in 1% (w/v) BSA in PBS and applied to the cells for 1 h at 37°C. The primary antibody was removed by washing the cells with PBS, and CF594-conjugated goat anti-mouse IgG1(γ1) (Biotium, #20249) was applied at a concentration of 2 µg/ml, together with DAPI (a final concentration of 1 µg/ml). Cells were incubated for 40 min at RT. Finally, the prepared coverslips were washed with PBS and rinsed with Milli-Q H_2_O before being mounted on a glass slide with a drop of VectorShield (Vector Laboratories, #H-1000). The samples were observed using a 2D array scanning laser confocal microscope (Infinity 3, VisiTech) using 60x/1.4 Plan Apo objective lens (Nikon). Each image comprises 35 serial images with 0.3 µm intervals along the Z-axis taken to span the full thickness of the cell. Images were Z-projected (maximum intensity), cropped and combined using Fiji (Schindelin et al., 2012). A quantile-quantile plot (q-q plot) of the obtained data showed that the data distribution was not parametric. Therefore, we used Kruskal–Wallis test and conducted a post hoc Dunn’s test for pairwise comparisons.

## Supplementary Material

Supplementary information

## References

[BIO059056C1] Abremski, K. and Hoess, R. (1984). Bacteriophage P1 site-specific recombination. Purification and properties of the Cre recombinase protein. *J. Biol. Chem.* 259, 1509-1514. 6319400

[BIO059056C2] Bodnar, A. G., Ouellette, M., Frolkis, M., Holt, S. E., Chiu, C. P., Morin, G. B., Harley, C. B., Shay, J. W., Lichtsteiner, S. and Wright, W. E. (1998). Extension of life-span by introduction of telomerase into normal human cells. *Science* 279, 349-352. 10.1126/science.279.5349.349 9454332

[BIO059056C3] Casanova, E., Fehsenfeld, S., Lemberger, T., Shimshek, D. R., Sprengel, R. and Mantamadiotis, T. (2002). ER-based double iCre fusion protein allows partial recombination in forebrain. *Genesis* 34, 208-214. 10.1002/gene.10153 12395386

[BIO059056C4] Cong, L., Ran, F. A., Cox, D., Lin, S., Barretto, R., Habib, N., Hsu, P. D., Wu, X., Jiang, W., Marraffini, L. A. et al. (2013). Multiplex genome engineering using CRISPR/Cas systems. *Science* 339, 819-823. 10.1126/science.1231143 23287718 PMC3795411

[BIO059056C5] Das, A. T., Tenenbaum, L. and Berkhout, B. (2016). Tet-on systems for doxycycline-inducible gene expression. *Curr. Gene Ther.* 16, 156-167. 10.2174/1566523216666160524144041 27216914 PMC5070417

[BIO059056C6] Feil, R., Wagner, J., Metzger, D. and Chambon, P. (1997). Regulation of Cre recombinase activity by mutated estrogen receptor ligand-binding domains. *Biochem. Biophys. Res. Commun.* 237, 752-757. 10.1006/bbrc.1997.7124 9299439

[BIO059056C7] Hoess, R. H., Ziese, M. and Sternberg, N. (1982). P1 site-specific recombination: nucleotide sequence of the recombining sites. *Proc. Natl. Acad. Sci. USA* 79, 3398-3402. 10.1073/pnas.79.11.3398 6954485 PMC346427

[BIO059056C8] Jiang, X. R., Jimenez, G., Chang, E., Frolkis, M., Kusler, B., Sage, M., Beeche, M., Bodnar, A. G., Wahl, G. M., Tlsty, T. D. et al. (1999). Telomerase expression in human somatic cells does not induce changes associated with a transformed phenotype. *Nat. Genet.* 21, 111-114. 10.1038/5056 9916802

[BIO059056C9] Katoh, Y., Michisaka, S., Nozaki, S., Funabashi, T., Hirano, T., Takei, R. and Nakayama, K. (2017). Practical method for targeted disruption of cilia-related genes by using CRISPR/Cas9-mediated, homology-independent knock-in system. *Mol. Biol. Cell* 28, 898-906. 10.1091/mbc.e17-01-0051 28179459 PMC5385939

[BIO059056C10] Kotin, R. M., Linden, R. M. and Berns, K. I. (1992). Characterization of a preferred site on human chromosome 19q for integration of adeno-associated virus DNA by non-homologous recombination. *EMBO J.* 11, 5071-5078. 10.1002/j.1460-2075.1992.tb05614.x 1334463 PMC556985

[BIO059056C11] Laemmli, U. K. (1970). Cleavage of structural proteins during the assembly of the head of bacteriophage T4. *Nature* 227, 680-685. 10.1038/227680a0 5432063

[BIO059056C12] Lakso, M., Sauer, B., Mosinger, B., Jr., Lee, E. J., Manning, R. W., Yu, S. H., Mulder, K. L. and Westphal, H. (1992). Targeted oncogene activation by site-specific recombination in transgenic mice. *Proc. Natl. Acad. Sci. USA* 89, 6232-6236. 10.1073/pnas.89.14.6232 1631115 PMC49474

[BIO059056C13] Loew, R., Heinz, N., Hampf, M., Bujard, H. and Gossen, M. (2010). Improved Tet-responsive promoters with minimized background expression. *BMC Biotechnol.* 10, 81. 10.1186/1472-6750-10-81 21106052 PMC3002914

[BIO059056C14] Mali, P., Yang, L., Esvelt, K. M., Aach, J., Guell, M., DiCarlo, J. E., Norville, J. E. and Church, G. M. (2013). RNA-guided human genome engineering via Cas9. *Science* 339, 823-826. 10.1126/science.1232033 23287722 PMC3712628

[BIO059056C15] Metzger, D., Clifford, J., Chiba, H. and Chambon, P. (1995). Conditional site-specific recombination in mammalian cells using a ligand-dependent chimeric Cre recombinase. *Proc. Natl. Acad. Sci. USA* 92, 6991-6995. 10.1073/pnas.92.15.6991 7624356 PMC41457

[BIO059056C16] Miyamoto, T., Hosoba, K., Ochiai, H., Royba, E., Izumi, H., Sakuma, T., Yamamoto, T., Dynlacht, B. D. and Matsuura, S. (2015). The microtubule-depolymerizing activity of a mitotic kinesin protein KIF2A drives primary cilia disassembly coupled with cell proliferation. *Cell Rep* 10, 664-673. 10.1016/j.celrep.2015.01.003 25660017 PMC5099117

[BIO059056C17] Natsume, T., Kiyomitsu, T., Saga, Y. and Kanemaki, M. T. (2016). Rapid protein depletion in human cells by auxin-inducible degron tagging with short homology donors. *Cell Rep* 15, 210-218. 10.1016/j.celrep.2016.03.001 27052166

[BIO059056C18] Ogata, T., Kozuka, T. and Kanda, T. (2003). Identification of an insulator in AAVS1, a preferred region for integration of adeno-associated virus DNA. *J. Virol.* 77, 9000-9007. 10.1128/JVI.77.16.9000-9007.2003 12885916 PMC167236

[BIO059056C19] Orban, P. C., Chui, D. and Marth, J. D. (1992). Tissue- and site-specific DNA recombination in transgenic mice. *Proc. Natl. Acad. Sci. USA* 89, 6861-6865. 10.1073/pnas.89.15.6861 1495975 PMC49604

[BIO059056C20] Rogakou, E. P., Pilch, D. R., Orr, A. H., Ivanova, V. S. and Bonner, W. M. (1998). DNA double-stranded breaks induce histone H2AX phosphorylation on serine 139. *J. Biol. Chem.* 273, 5858-5868. 10.1074/jbc.273.10.5858 9488723

[BIO059056C21] Sauer, B. and Henderson, N. (1988). Site-specific DNA recombination in mammalian cells by the Cre recombinase of bacteriophage P1. *Proc. Natl. Acad. Sci. USA* 85, 5166-5170. 10.1073/pnas.85.14.5166 2839833 PMC281709

[BIO059056C22] Schindelin, J., Arganda-Carreras, I., Frise, E., Kaynig, V., Longair, M., Pietzsch, T., Preibisch, S., Rueden, C., Saalfeld, S., Schmid, B. et al. (2012). Fiji: an open-source platform for biological-image analysis. *Nat. Methods* 9, 676-682. 10.1038/nmeth.2019 22743772 PMC3855844

[BIO059056C23] Schmidt, E. E., Taylor, D. S., Prigge, J. R., Barnett, S. and Capecchi, M. R. (2000). Illegitimate Cre-dependent chromosome rearrangements in transgenic mouse spermatids. *Proc. Natl. Acad. Sci. USA* 97, 13702-13707. 10.1073/pnas.240471297 11087830 PMC17639

[BIO059056C24] Sengupta, R., Mendenhall, A., Sarkar, N., Mukherjee, C., Afshari, A., Huang, J. and Lu, B. (2017). Viral Cre-LoxP tools aid genome engineering in mammalian cells. *J Biol Eng* 11, 45. 10.1186/s13036-017-0087-y 29204184 PMC5702101

[BIO059056C25] Senmatsu, S., Asada, R., Oda, A., Hoffman, C. S., Ohta, K. and Hirota, K. (2021). lncRNA transcription induces meiotic recombination through chromatin remodelling in fission yeast. *Commun. Biol.* 4, 295. 10.1038/s42003-021-01798-8 33674718 PMC7935937

[BIO059056C26] Silver, D. P. and Livingston, D. M. (2001). Self-excising retroviral vectors encoding the Cre recombinase overcome Cre-mediated cellular toxicity. *Mol. Cell* 8, 233-243. 10.1016/S1097-2765(01)00295-7 11511376

[BIO059056C27] Smith, J. R., Maguire, S., Davis, L. A., Alexander, M., Yang, F., Chandran, S., ffrench-Constant, C. and Pedersen, R. A. (2008). Robust, persistent transgene expression in human embryonic stem cells is achieved with AAVS1-targeted integration. *Stem Cells* 26, 496-504. 18024421 10.1634/stemcells.2007-0039

[BIO059056C28] Sternberg, N. and Hamilton, D. (1981). Bacteriophage P1 site-specific recombination. I. Recombination between loxP sites. *J. Mol. Biol.* 150, 467-486. 6276557 10.1016/0022-2836(81)90375-2

[BIO059056C29] Uhl, E. W. and Warner, N. J. (2015). Mouse models as predictors of human responses: evolutionary medicine. *Curr Pathobiol Rep* 3, 219-223. 10.1007/s40139-015-0086-y 26246962 PMC4522464

[BIO059056C30] Van Duyne, G. D. (2001). A structural view of cre-loxp site-specific recombination. *Annu. Rev. Biophys. Biomol. Struct.* 30, 87-104. 10.1146/annurev.biophys.30.1.87 11340053

[BIO059056C31] Vara, J. A., Portela, A., Ortin, J. and Jimenez, A. (1986). Expression in mammalian cells of a gene from Streptomyces alboniger conferring puromycin resistance. *Nucleic Acids Res.* 14, 4617-4624. 10.1093/nar/14.11.4617 3714487 PMC311469

[BIO059056C32] Wang, X. (2009). Cre transgenic mouse lines. *Methods Mol. Biol.* 561, 265-273. 10.1007/978-1-60327-019-9_17 19504077

[BIO059056C33] Weinrich, S. L., Pruzan, R., Ma, L., Ouellette, M., Tesmer, V. M., Holt, S. E., Bodnar, A. G., Lichtsteiner, S., Kim, N. W., Trager, J. B. et al. (1997). Reconstitution of human telomerase with the template RNA component hTR and the catalytic protein subunit hTRT. *Nat. Genet.* 17, 498-502. 10.1038/ng1297-498 9398860

[BIO059056C34] Zhang, Y., Riesterer, C., Ayrall, A. M., Sablitzky, F., Littlewood, T. D. and Reth, M. (1996). Inducible site-directed recombination in mouse embryonic stem cells. *Nucleic Acids Res.* 24, 543-548. 10.1093/nar/24.4.543 8604292 PMC145690

[BIO059056C35] Zhou, X., Vink, M., Klaver, B., Berkhout, B. and Das, A. T. (2006). Optimization of the Tet-On system for regulated gene expression through viral evolution. *Gene Ther.* 13, 1382-1390. 10.1038/sj.gt.3302780 16724096

