# Supplementary material for: Construction of a human hTERT RPE-1 cell line with inducible Cre for editing of endogenous genes
Source: Biol Open. 2022 Feb 16;11(2):bio059056. doi: 10.1242/bio.059056 (PMC8864296; doi:10.1242/bio.059056)
Supplement: Supplementary information [file biolopen-11-059056-s1.pdf]

Supplementary Fig. 1A

The DNA sequence encoding ER^{T2}CreER^{T2} at *AAVS1* locus of clone 19

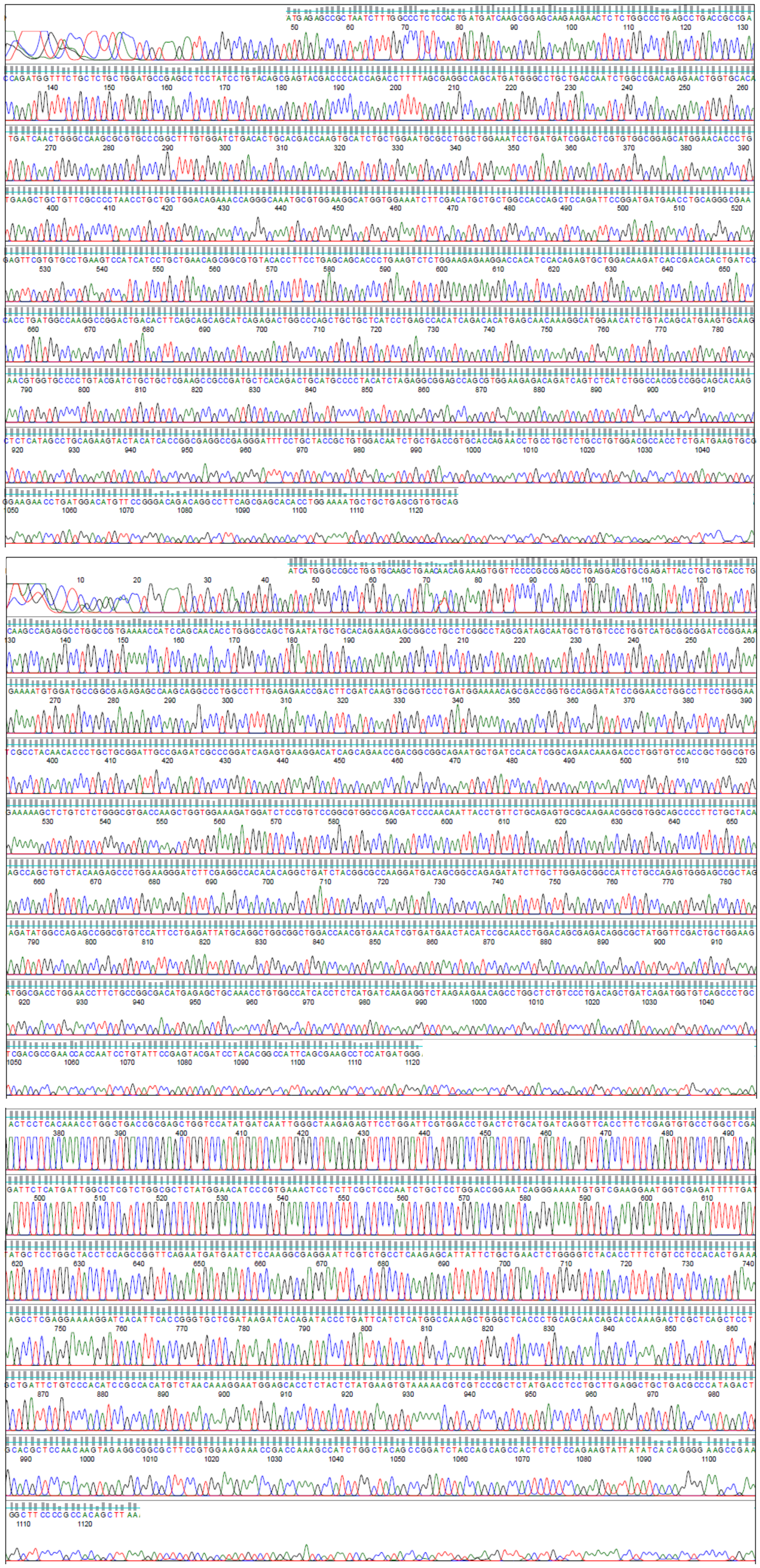

Supplementary Fig. 1B

The DNA sequence encoding $ER^{T2}CreER^{T2}$ at *AAVS1* locus of clone 65

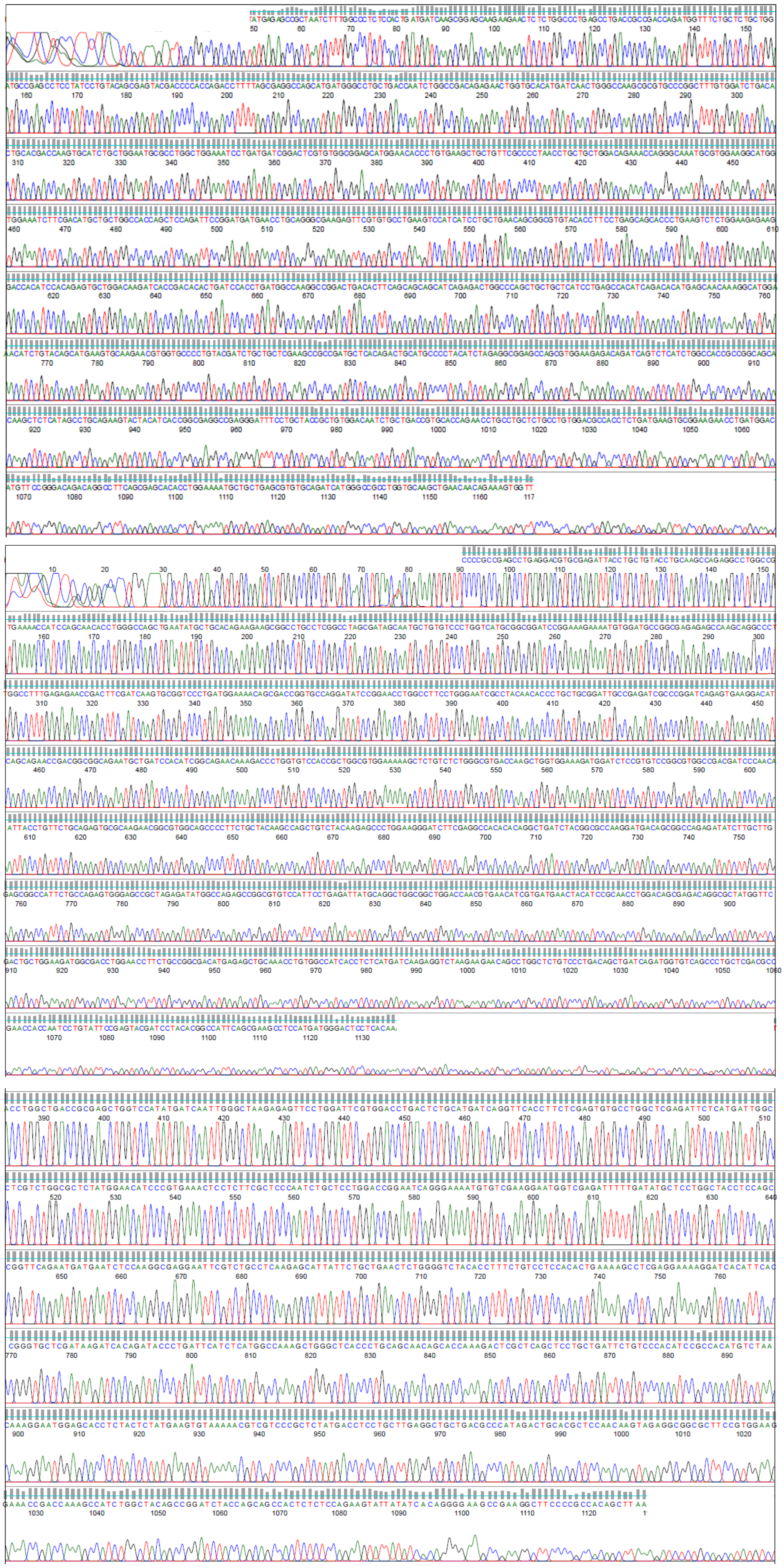

Biology Open • Supplementary information

Supplementary Fig. 1C

```
ERT2CreERT2      ATGAGAGCCGCTAATCTTTGGCCCTCTCCACTGATGATCAAGCGGAGCAAGAAGAACTCTCTGGCCCTGAGCCTGACCGCCGACCAGATGGTTTCTGCTCTGCTGGATGCCGAGCCTCCT 120
Clone19          ATGAGAGCCGCTAATCTTTGGCCCTCTCCACTGATGATCAAGCGGAGCAAGAAGAACTCTCTGGCCCTGAGCCTGACCGCCGACCAGATGGTTTCTGCTCTGCTGGATGCCGAGCCTCCT 120
clone65          ATGAGAGCCGCTAATCTTTGGCCCTCTCCACTGATGATCAAGCGGAGCAAGAAGAACTCTCTGGCCCTGAGCCTGACCGCCGACCAGATGGTTTCTGCTCTGCTGGATGCCGAGCCTCCT 120
                 ************************************************************************************************************************

ERT2CreERT2      ATCCTGTACAGCGAGTACGACCCCACCAGACCTTTTAGCGAGGCCAGCATGATGGGCCTGCTGACCAATCTGGCCGACAGAGAACTGGTGCACATGATCAACTGGGCCAAGCGCGTGCCC 240
Clone19          ATCCTGTACAGCGAGTACGACCCCACCAGACCTTTTAGCGAGGCCAGCATGATGGGCCTGCTGACCAATCTGGCCGACAGAGAACTGGTGCACATGATCAACTGGGCCAAGCGCGTGCCC 240
clone65          ATCCTGTACAGCGAGTACGACCCCACCAGACCTTTTAGCGAGGCCAGCATGATGGGCCTGCTGACCAATCTGGCCGACAGAGAACTGGTGCACATGATCAACTGGGCCAAGCGCGTGCCC 240
                 ************************************************************************************************************************

ERT2CreERT2      GGCTTTGTGGATCTGACACTGCACGACCAAGTGCATCTGCTGGAATGCGCCTGGCTGGAAATCCTGATGATCGGACTCGTGTGGCGGAGCATGGAACACCCTGTGAAGCTGCTGTTCGCC 360
Clone19          GGCTTTGTGGATCTGACACTGCACGACCAAGTGCATCTGCTGGAATGCGCCTGGCTGGAAATCCTGATGATCGGACTCGTGTGGCGGAGCATGGAACACCCTGTGAAGCTGCTGTTCGCC 360
clone65          GGCTTTGTGGATCTGACACTGCACGACCAAGTGCATCTGCTGGAATGCGCCTGGCTGGAAATCCTGATGATCGGACTCGTGTGGCGGAGCATGGAACACCCTGTGAAGCTGCTGTTCGCC 360
                 ************************************************************************************************************************

ERT2CreERT2      CCTAACCTGCTGCTGGACAGAAACCAGGGCAAATGCGTGGAAGGCATGGTGGAAATCTTCGACATGCTGCTGGCCACCAGCTCCAGATTCCGGATGATGAACCTGCAGGGCGAAGAGTTC 480
Clone19          CCTAACCTGCTGCTGGACAGAAACCAGGGCAAATGCGTGGAAGGCATGGTGGAAATCTTCGACATGCTGCTGGCCACCAGCTCCAGATTCCGGATGATGAACCTGCAGGGCGAAGAGTTC 480
clone65          CCTAACCTGCTGCTGGACAGAAACCAGGGCAAATGCGTGGAAGGCATGGTGGAAATCTTCGACATGCTGCTGGCCACCAGCTCCAGATTCCGGATGATGAACCTGCAGGGCGAAGAGTTC 480
                 ************************************************************************************************************************

ERT2CreERT2      GTGTGCCTGAAGTCCATCATCCTGCTGAACAGCGGCGTGTACACCTTCCTGAGCAGCACCCTGAAGTCTCTGGAAGAGAAGGACCACATCCACAGAGTGCTGGACAAGATCACCGACACA 600
Clone19          GTGTGCCTGAAGTCCATCATCCTGCTGAACAGCGGCGTGTACACCTTCCTGAGCAGCACCCTGAAGTCTCTGGAAGAGAAGGACCACATCCACAGAGTGCTGGACAAGATCACCGACACA 600
clone65          GTGTGCCTGAAGTCCATCATCCTGCTGAACAGCGGCGTGTACACCTTCCTGAGCAGCACCCTGAAGTCTCTGGAAGAGAAGGACCACATCCACAGAGTGCTGGACAAGATCACCGACACA 600
                 ************************************************************************************************************************

ERT2CreERT2      CTGATCCACCTGATGGCCAAGGCCGGACTGACACTTCAGCAGCAGCATCAGAGACTGGCCCAGCTGCTGCTCATCCTGAGCCACATCAGACACATGAGCAACAAAGGCATGGAACATCTG 720
Clone19          CTGATCCACCTGATGGCCAAGGCCGGACTGACACTTCAGCAGCAGCATCAGAGACTGGCCCAGCTGCTGCTCATCCTGAGCCACATCAGACACATGAGCAACAAAGGCATGGAACATCTG 720
clone65          CTGATCCACCTGATGGCCAAGGCCGGACTGACACTTCAGCAGCAGCATCAGAGACTGGCCCAGCTGCTGCTCATCCTGAGCCACATCAGACACATGAGCAACAAAGGCATGGAACATCTG 720
                 ************************************************************************************************************************

ERT2CreERT2      TACAGCATGAAGTGCAAGAACGTGGTGCCCCTGTACGATCTGCTGCTCGAAGCCGCCGATGCTCACAGACTGCATGCCCCTACATCTAGAGGCGGAGCCAGCGTGGAAGAGACAGATCAG 840
Clone19          TACAGCATGAAGTGCAAGAACGTGGTGCCCCTGTACGATCTGCTGCTCGAAGCCGCCGATGCTCACAGACTGCATGCCCCTACATCTAGAGGCGGAGCCAGCGTGGAAGAGACAGATCAG 840
clone65          TACAGCATGAAGTGCAAGAACGTGGTGCCCCTGTACGATCTGCTGCTCGAAGCCGCCGATGCTCACAGACTGCATGCCCCTACATCTAGAGGCGGAGCCAGCGTGGAAGAGACAGATCAG 840
                 ************************************************************************************************************************

ERT2CreERT2      TCTCATCTGGCCACCGCCGGCAGCACAAGCTCTCATAGCCTGCAGAAGTACTACATCACCGGCGAGGCCGAGGGATTTCCTGCTACCGCTGTGGACAATCTGCTGACCGTGCACCAGAAC 960
Clone19          TCTCATCTGGCCACCGCCGGCAGCACAAGCTCTCATAGCCTGCAGAAGTACTACATCACCGGCGAGGCCGAGGGATTTCCTGCTACCGCTGTGGACAATCTGCTGACCGTGCACCAGAAC 960
clone65          TCTCATCTGGCCACCGCCGGCAGCACAAGCTCTCATAGCCTGCAGAAGTACTACATCACCGGCGAGGCCGAGGGATTTCCTGCTACCGCTGTGGACAATCTGCTGACCGTGCACCAGAAC 960
                 ************************************************************************************************************************

ERT2CreERT2      CTGCCTGCTCTGCCTGTGGACGCCACCTCTGATGAAGTGCGGAAGAACCTGATGGACATGTTCCGGGACAGACAGGCCTTCAGCGAGCACACCTGGAAAATGCTGCTGAGCGTGTGCAGA 1080
Clone19          CTGCCTGCTCTGCCTGTGGACGCCACCTCTGATGAAGTGCGGAAGAACCTGATGGACATGTTCCGGGACAGACAGGCCTTCAGCGAGCACACCTGGAAAATGCTGCTGAGCGTGTGCAGA 1080
clone65          CTGCCTGCTCTGCCTGTGGACGCCACCTCTGATGAAGTGCGGAAGAACCTGATGGACATGTTCCGGGACAGACAGGCCTTCAGCGAGCACACCTGGAAAATGCTGCTGAGCGTGTGCAGA 1080
                 ************************************************************************************************************************

ERT2CreERT2      TCATGGGCCGCCTGGTGCAAGCTGAACAACAGAAAGTGGTTCCCCGCCGAGCCTGAGGACGTGCGAGATTACCTGCTGTACCTGCAAGCCAGAGGCCTGGCCGTGAAAACCATCCAGCAA 1200
Clone19          TCATGGGCCGCCTGGTGCAAGCTGAACAACAGAAAGTGGTTCCCCGCCGAGCCTGAGGACGTGCGAGATTACCTGCTGTACCTGCAAGCCAGAGGCCTGGCCGTGAAAACCATCCAGCAA 1200
clone65          TCATGGGCCGCCTGGTGCAAGCTGAACAACAGAAAGTGGTTCCCCGCCGAGCCTGAGGACGTGCGAGATTACCTGCTGTACCTGCAAGCCAGAGGCCTGGCCGTGAAAACCATCCAGCAA 1200
                 ************************************************************************************************************************

ERT2CreERT2      CACCTGGGCCAGCTGAATATGCTGCACAGAAGAAGCGGCCTGCCTCGGCCTAGCGAtAGCAATGCTGTGTCCCTGGTCATGCGGCGGATCCGGAAAGAAAATGTGGATGCCGGCGAGAGA 1320
Clone19          CACCTGGGCCAGCTGAATATGCTGCACAGAAGAAGCGGCCTGCCTCGGCCTAGCGAtAGCAATGCTGTGTCCCTGGTCATGCGGCGGATCCGGAAAGAAAATGTGGATGCCGGCGAGAGA 1320
clone65          CACCTGGGCCAGCTGAATATGCTGCACAGAAGAAGCGGCCTGCCTCGGCCTAGCGAtAGCAATGCTGTGTCCCTGGTCATGCGGCGGATCCGGAAAGAAAATGTGGATGCCGGCGAGAGA 1320
                 ************************************************************************************************************************

ERT2CreERT2      GCCAAGCAGGCCCTGGCCTTTGAGAGAACCGACTTCGATCAAGTGCGGTCCCTGATGGAAAACAGCGACCGGTGCCAGGATATCCGGAACCTGGCCTTCCTGGGAATCGCCTACAACACC 1440
Clone19          GCCAAGCAGGCCCTGGCCTTTGAGAGAACCGACTTCGATCAAGTGCGGTCCCTGATGGAAAACAGCGACCGGTGCCAGGATATCCGGAACCTGGCCTTCCTGGGAATCGCCTACAACACC 1440
clone65          GCCAAGCAGGCCCTGGCCTTTGAGAGAACCGACTTCGATCAAGTGCGGTCCCTGATGGAAAACAGCGACCGGTGCCAGGATATCCGGAACCTGGCCTTCCTGGGAATCGCCTACAACACC 1440
                 ************************************************************************************************************************

ERT2CreERT2      CTGCTGCGGATTGCCGAGATCGCCCGGATCAGAGTGAAGGACATCAGCAGAACCGACGGCGGCAGAATGCTGATCCACATCGGCAGAACAAAGACCCTGGTGTCCACCGCTGGCGTGGAA 1560
Clone19          CTGCTGCGGATTGCCGAGATCGCCCGGATCAGAGTGAAGGACATCAGCAGAACCGACGGCGGCAGAATGCTGATCCACATCGGCAGAACAAAGACCCTGGTGTCCACCGCTGGCGTGGAA 1560
clone65          CTGCTGCGGATTGCCGAGATCGCCCGGATCAGAGTGAAGGACATCAGCAGAACCGACGGCGGCAGAATGCTGATCCACATCGGCAGAACAAAGACCCTGGTGTCCACCGCTGGCGTGGAA 1560
                 ************************************************************************************************************************

ERT2CreERT2      AAAGctCTGTCTCTGGGCGTGACCAAGCTGGTGGAAAGATGGATCTCCGTGTCCGGCGTGGCCGACGATCCCAACAATTACCTGTTCTGCAGAGTGCGCAAGAACGGCGTGGCAGCCCCT 1680
Clone19          AAAGctCTGTCTCTGGGCGTGACCAAGCTGGTGGAAAGATGGATCTCCGTGTCCGGCGTGGCCGACGATCCCAACAATTACCTGTTCTGCAGAGTGCGCAAGAACGGCGTGGCAGCCCCT 1680
clone65          AAAGctCTGTCTCTGGGCGTGACCAAGCTGGTGGAAAGATGGATCTCCGTGTCCGGCGTGGCCGACGATCCCAACAATTACCTGTTCTGCAGAGTGCGCAAGAACGGCGTGGCAGCCCCT 1680
                 ************************************************************************************************************************

ERT2CreERT2      TCTGCTACAAGCCAGCTGTCTACAAGAGCCCTGGAAGGGATCTTCGAGGCCACACACAGGCTGATCTACGGCGCCAAGGATGACAGCGGCCAGAGATATCTTGCTTGGAGCGGCCATTCT 1800
Clone19          TCTGCTACAAGCCAGCTGTCTACAAGAGCCCTGGAAGGGATCTTCGAGGCCACACACAGGCTGATCTACGGCGCCAAGGATGACAGCGGCCAGAGATATCTTGCTTGGAGCGGCCATTCT 1800
clone65          TCTGCTACAAGCCAGCTGTCTACAAGAGCCCTGGAAGGGATCTTCGAGGCCACACACAGGCTGATCTACGGCGCCAAGGATGACAGCGGCCAGAGATATCTTGCTTGGAGCGGCCATTCT 1800
                 ************************************************************************************************************************

ERT2CreERT2      GCCAGAGTGGGAGCCGCTAGAGATATGGCCAGAGCCGGCGTGTCCATTCCTGAGATTATGCAGGCTGGCGGCTGGACCAACGTGAACATCGTGATGAACTACATCCGCAACCTGGACAGC 1920
Clone19          GCCAGAGTGGGAGCCGCTAGAGATATGGCCAGAGCCGGCGTGTCCATTCCTGAGATTATGCAGGCTGGCGGCTGGACCAACGTGAACATCGTGATGAACTACATCCGCAACCTGGACAGC 1920
clone65          GCCAGAGTGGGAGCCGCTAGAGATATGGCCAGAGCCGGCGTGTCCATTCCTGAGATTATGCAGGCTGGCGGCTGGACCAACGTGAACATCGTGATGAACTACATCCGCAACCTGGACAGC 1920
                 ************************************************************************************************************************

ERT2CreERT2      GAGACAGGCGCTATGGTTCGACTGCTGGAAGATGGCGACCTGGAACCTTCTGCCGGCGACATGAGAGCTGCAAACCTGTGGCCATCACCTCTCATGATCAAGAGGTCTAAGAAGAACAGC 2040
Clone19          GAGACAGGCGCTATGGTTCGACTGCTGGAAGATGGCGACCTGGAACCTTCTGCCGGCGACATGAGAGCTGCAAACCTGTGGCCATCACCTCTCATGATCAAGAGGTCTAAGAAGAACAGC 2040
clone65          GAGACAGGCGCTATGGTTCGACTGCTGGAAGATGGCGACCTGGAACCTTCTGCCGGCGACATGAGAGCTGCAAACCTGTGGCCATCACCTCTCATGATCAAGAGGTCTAAGAAGAACAGC 2040
                 ************************************************************************************************************************

ERT2CreERT2      CTGGCTCTGTCCCTGACAGCTGATCAGATGGTGTCAGCCCTGCTCGACGCCGAACCACCAATCCTGTATTCCGAGTACGATCCTACACGGCCATTCAGCGAAGCCTCCATGATGGGACTC 2160
Clone19          CTGGCTCTGTCCCTGACAGCTGATCAGATGGTGTCAGCCCTGCTCGACGCCGAACCACCAATCCTGTATTCCGAGTACGATCCTACACGGCCATTCAGCGAAGCCTCCATGATGGGACTC 2160
clone65          CTGGCTCTGTCCCTGACAGCTGATCAGATGGTGTCAGCCCTGCTCGACGCCGAACCACCAATCCTGTATTCCGAGTACGATCCTACACGGCCATTCAGCGAAGCCTCCATGATGGGACTC 2160
                 ************************************************************************************************************************

ERT2CreERT2      CTCACAAACCTGGCTGACCGCGAGCTGGTCCATATGATCAATTGGGCTAAGAGAGTTCCTGGATTCGTGGACCTGACTCTGCATGATCAGGTTCACCTTCTCGAGTGTGCCTGGCTCGAG 2280
Clone19          CTCACAAACCTGGCTGACCGCGAGCTGGTCCATATGATCAATTGGGCTAAGAGAGTTCCTGGATTCGTGGACCTGACTCTGCATGATCAGGTTCACCTTCTCGAGTGTGCCTGGCTCGAG 2280
clone65          CTCACAAACCTGGCTGACCGCGAGCTGGTCCATATGATCAATTGGGCTAAGAGAGTTCCTGGATTCGTGGACCTGACTCTGCATGATCAGGTTCACCTTCTCGAGTGTGCCTGGCTCGAG 2280
                 ************************************************************************************************************************

ERT2CreERT2      ATTCTCATGATTGGCCTCGTCTGGCGCTCTATGGAACATCCCGTGAAACTCCTCTTCGCTCCCAATCTGCTCCTGGACCGGAATCAGGGAAAATGTGTCGAAGGAATGGTCGAGATTTTT 2400
Clone19          ATTCTCATGATTGGCCTCGTCTGGCGCTCTATGGAACATCCCGTGAAACTCCTCTTCGCTCCCAATCTGCTCCTGGACCGGAATCAGGGAAAATGTGTCGAAGGAATGGTCGAGATTTTT 2400
clone65          ATTCTCATGATTGGCCTCGTCTGGCGCTCTATGGAACATCCCGTGAAACTCCTCTTCGCTCCCAATCTGCTCCTGGACCGGAATCAGGGAAAATGTGTCGAAGGAATGGTCGAGATTTTT 2400
                 ************************************************************************************************************************

ERT2CreERT2      GATATGCTCCTGGCTACCTCCAGCCGGTTCAGAATGATGAATCTCCAAGGCGAGGAATTCGTCTGCCTCAAGAGCATTATTCTGCTGAACTCTGGGGTCTACACCTTTCTGTCCTCCACA 2520
Clone19          GATATGCTCCTGGCTACCTCCAGCCGGTTCAGAATGATGAATCTCCAAGGCGAGGAATTCGTCTGCCTCAAGAGCATTATTCTGCTGAACTCTGGGGTCTACACCTTTCTGTCCTCCACA 2520
clone65          GATATGCTCCTGGCTACCTCCAGCCGGTTCAGAATGATGAATCTCCAAGGCGAGGAATTCGTCTGCCTCAAGAGCATTATTCTGCTGAACTCTGGGGTCTACACCTTTCTGTCCTCCACA 2520
                 ************************************************************************************************************************

ERT2CreERT2      CTGAAAAGCCTCGAGGAAAAGGATCACATTCACCGGGTGCTCGATAAGATCACAGATACCCTGATTCATCTCATGGCCAAAGCTGGGCTCACCCTGCAGCAACAGCACCAAAGACTCGCT 2640
Clone19          CTGAAAAGCCTCGAGGAAAAGGATCACATTCACCGGGTGCTCGATAAGATCACAGATACCCTGATTCATCTCATGGCCAAAGCTGGGCTCACCCTGCAGCAACAGCACCAAAGACTCGCT 2640
clone65          CTGAAAAGCCTCGAGGAAAAGGATCACATTCACCGGGTGCTCGATAAGATCACAGATACCCTGATTCATCTCATGGCCAAAGCTGGGCTCACCCTGCAGCAACAGCACCAAAGACTCGCT 2640
                 ************************************************************************************************************************

ERT2CreERT2      CAGCTCCTGCTGATTCTGTCCCACATCCGCCACATGTCTAACAAAGGAATGGAGCACCTCTACTCTATGAAGTGTAAAAACGTCGTCCCGCTCTATGACCTCCTGCTTGAGGCTGCTGAC 2760
Clone19          CAGCTCCTGCTGATTCTGTCCCACATCCGCCACATGTCTAACAAAGGAATGGAGCACCTCTACTCTATGAAGTGTAAAAACGTCGTCCCGCTCTATGACCTCCTGCTTGAGGCTGCTGAC 2760
clone65          CAGCTCCTGCTGATTCTGTCCCACATCCGCCACATGTCTAACAAAGGAATGGAGCACCTCTACTCTATGAAGTGTAAAAACGTCGTCCCGCTCTATGACCTCCTGCTTGAGGCTGCTGAC 2760
                 ************************************************************************************************************************

ERT2CreERT2      GCCCATAGACTGCACGCTCCAACAAGTAGAGGCGGCGCTTCCGTGGAAGAAACCGACCAAAGCCATCTGGCTACAGCCGGATCTACCAGCAGCCACTCTCTCCAGAAGTATTATATCACA 2880
Clone19          GCCCATAGACTGCACGCTCCAACAAGTAGAGGCGGCGCTTCCGTGGAAGAAACCGACCAAAGCCATCTGGCTACAGCCGGATCTACCAGCAGCCACTCTCTCCAGAAGTATTATATCACA 2880
clone65          GCCCATAGACTGCACGCTCCAACAAGTAGAGGCGGCGCTTCCGTGGAAGAAACCGACCAAAGCCATCTGGCTACAGCCGGATCTACCAGCAGCCACTCTCTCCAGAAGTATTATATCACA 2880
                 ************************************************************************************************************************

ERT2CreERT2      GGGGAAGCCGAAGGCTTCCCCGCCACAGCTTAA  2913
Clone19          GGGGAAGCCGAAGGCTTCCCCGCCACAGCTTAA  2913
clone65          GGGGAAGCCGAAGGCTTCCCCGCCACAGCTTAA  2913
                 *********************************
```

Fig. S1. Clone 19 and Clone 65 carry an intact DNA fragment encoding ER^{T2}-Cre-ER^{T2}. (A) Three sequencing chromatograms that cover clone 19 *AAVS1* locus encoding ER^{T2}-Cre-ER^{T2}. (B) Three sequencing chromatograms that cover clone 65 *AAVS1* locus encoding ER^{T2}-Cre-ER^{T2}. (C) Sequence alignment of the intended DNA fragment encoding ER^{T2}-Cre-ER^{T2}, clone 19 *AAVS1* locus encoding ER^{T2}-Cre-ER^{T2}, and clone 65 *AAVS1* locus encoding ER^{T2}-Cre-ER^{T2}.

Biology Open • Supplementary information

Supplementary Figure 2

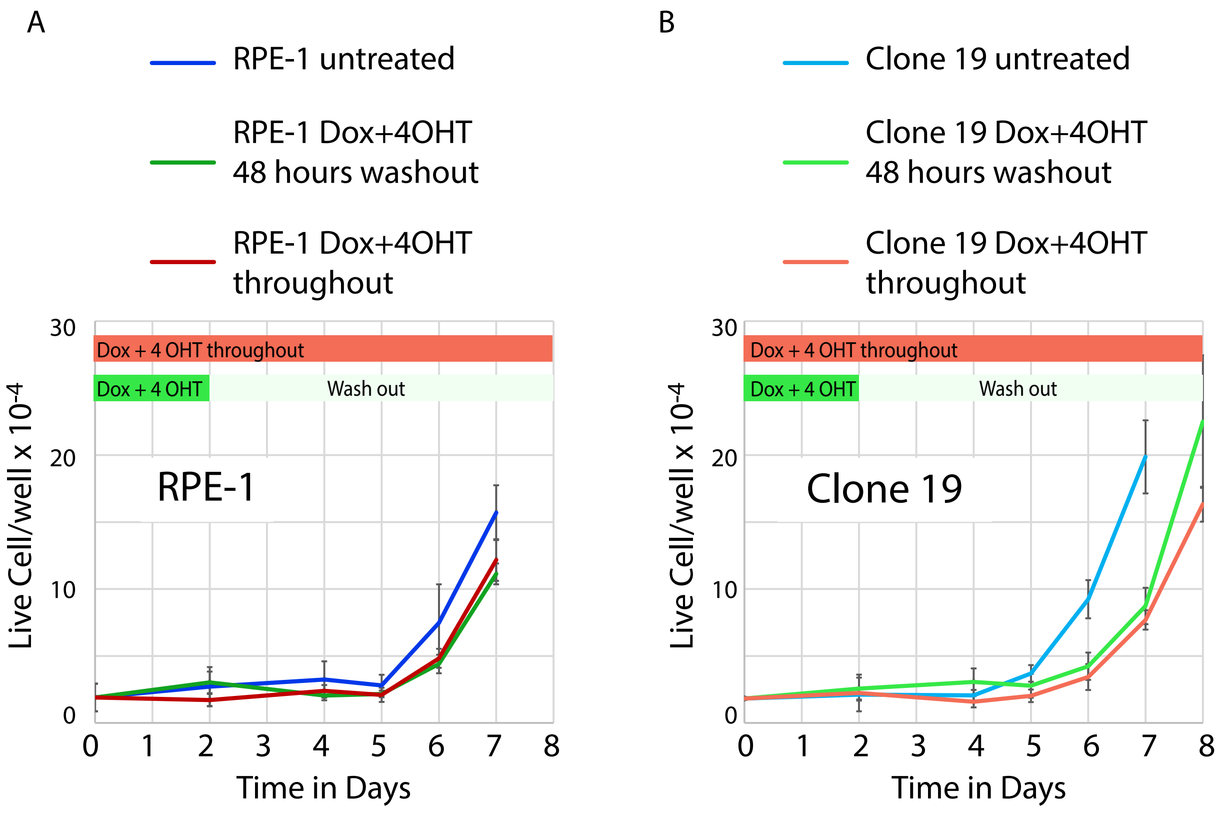

Biology Open • Supplementary information

Fig. S2. The cell count data used to produce Figure 4. Proliferation profiles of RPE1 and clone 19 cells with or without doxycycline and 4-OHT treatment. The data presented in this figure was used to generate Figure 4 (A)-(C). A relatively small number of cells (2 x 10^3 cells) were seeded per well in a 6-well plate to allow observation of proliferation without passaging for up to 8 days. (A) Proliferation rates of RPE1 cells without doxycycline and 4-OHT treatment (blue), RPE1 cells treated for 48 hours with doxycycline and 4-OHT before replacement into fresh media (green), and RPE1 cells cultured in the presence of doxycycline and 4-OHT throughout the experiment (red). (B) Proliferation rates of clone 19 with or without doxycycline and 4-OHT treatment were measured as in (A). For the samples treated with doxycycline and 4-OHT (green and red), cell counts were made for an additional day to reveal the growth recovery of these samples. All experiments, n=3; error bars represent standard deviation (SD).
